# A retrospective analysis of multiparametric ultrasound features in chromophobe renal cell carcinoma

**DOI:** 10.3389/fonc.2025.1587714

**Published:** 2025-08-07

**Authors:** Xiaoying Lin, Yucheng Lin, Lingpeng Tang, Ting Hu, Songsong Wu

**Affiliations:** ^1^ Shengli Clinical Medical College of Fujian Medical University, Fujian Medical University, Fuzhou, China; ^2^ Department of Ultrasonography, Shengli Clinical Medical College of Fujian Medical University, Fujian Provincial Hospital, Fuzhou University Affiliated Provincial Hospital, Fuzhou, China; ^3^ Department of Medical Ultrasonics, Fujian Maternity and Child Health Hospital, College of Clinical Medicine for Obstetrics & Gynecology and Pediatrics, Fujian Medical University, Fuzhou, China

**Keywords:** chromophobe renal cell carcinoma, 3D slicer, conventional ultrasound, contrast-enhanced ultrasound, echo intensity values

## Abstract

**Objective:**

To analyze the echo intensity values, conventional ultrasound features, and contrast-enhanced ultrasound (CEUS) characteristics of chromophobe renal cell carcinoma (chRCC), aiming to provide a valuable reference for the non-invasive clinical diagnosis of chRCC.

**Methods:**

This retrospective study included 52 patients with pathologically proven chRCC at Fujian Provincial Hospital between June 2010 and January 2024, all of whom underwent ultrasound examinations prior to treatment. Two ultrasound specialists assessed the imaging features of each tumor, and 3D Slicer software was utilized to measure the echo intensity values of the renal cortex, renal mass, and renal sinus from the same ultrasound scan plane.

**Results:**

51.9% of the patients included in this study were male, with an average age of 52.8 years. Quantitative echo intensity measurements showed that only 23.1% (12/52) of the tumors had lower echo intensity compared to the renal cortex, whereas 75.0% (39/52) had values between those of the renal cortex and renal sinus. The median tumor-to-renal cortex echo intensity ratio was 1.18, with the first and third quartiles (Q1, Q3) being 1.01 and 1.78, respectively. Conventional ultrasound analysis revealed that 80.8% (42/52) of the tumors exhibited a regular shape, while 78.9% (41/52) were completely or predominantly solid. In CEUS, 48.4% (15/31) of the tumors exhibited slow wash-in, while 77.4% (24/31) showed fast wash-out. Furthermore, 71.0% (22/31) demonstrated homogeneous peak enhancement, and 61.3% (19/31) displayed perilesional rim-like enhancement (PRE).

**Conclusion:**

The combination of conventional ultrasound features and CEUS characteristics of chRCC with quantitative echo intensity analysis enhances diagnostic objectivity and holds promise for non-invasive preoperative differentiation of RCC subtypes.

## 1 Introduction

Chromophobe renal cell carcinoma (chRCC) is the third most prevalent subtype RCC, accounting for approximately 5% of all RCC cases (1). It tends to be less invasive, with a five-year survival rate of around 90% (2). The clinical behavior of RCC subtypes varies significantly, and multiple treatment options are available (3). The 2019 European Association of Urology guidelines advocate for partial nephrectomy in clinical stage T1 RCC (T1a or T1b). In addition, growing evidence supports active surveillance or ablation therapy for T1a RCC (4). Compared to radical nephrectomy, partial nephrectomy is associated with a lower risk of postoperative cardiovascular events (5). Therefore, it is crucial to detect and treat renal cell carcinoma at an early stage through renal imaging. Early detection is not only essential for the clinical management of kidney disease patients, but also significantly impacts their prognosis (6). Contrast-enhanced ultrasound (CEUS) excels in showing vascular distribution and lesion perfusion (7), with higher sensitivity in diagnosing renal tumors compared to contrast-enhanced magnetic resonance imaging (CEMR) (8). Previous ultrasound studies on chRCC have primarily focused on comparing it with other renal tumor subtypes (9, 10), without providing a comprehensive overview of the ultrasound characteristics specific to chRCC. 3D Slicer (http://www.slicer.org) is an open-source software for medical image analysis and research, providing a robust platform for multi-modal data processing and visualization. It has recently become a widely used tool in medical image processing (11). Along with retrospective ultrasound image analysis, we employed 3D Slicer software to assess the echo intensity values of chRCC. While previous studies have demonstrated the potential of echo intensity values in evaluating skeletal muscle quality and function (12), their application in renal tumors remains unexplored. By leveraging 3D Slicer for quantitative echo analysis, we aimed to provide a more objective assessment, further enriching the ultrasound characterization of chRCC.

This study included 52 pathologically confirmed chRCC patients who underwent pre-treatment ultrasound, representing the largest cohort focused on chRCC ultrasound features. We conducted a multi-parameter, multi-dimensional analysis from three perspectives: echo intensity values, conventional ultrasound, and CEUS, aiming to provide a reference for future non-invasive diagnosis of chRCC.

## 2 Materials and methods

### 2.1 Patients

**Figure 1:**
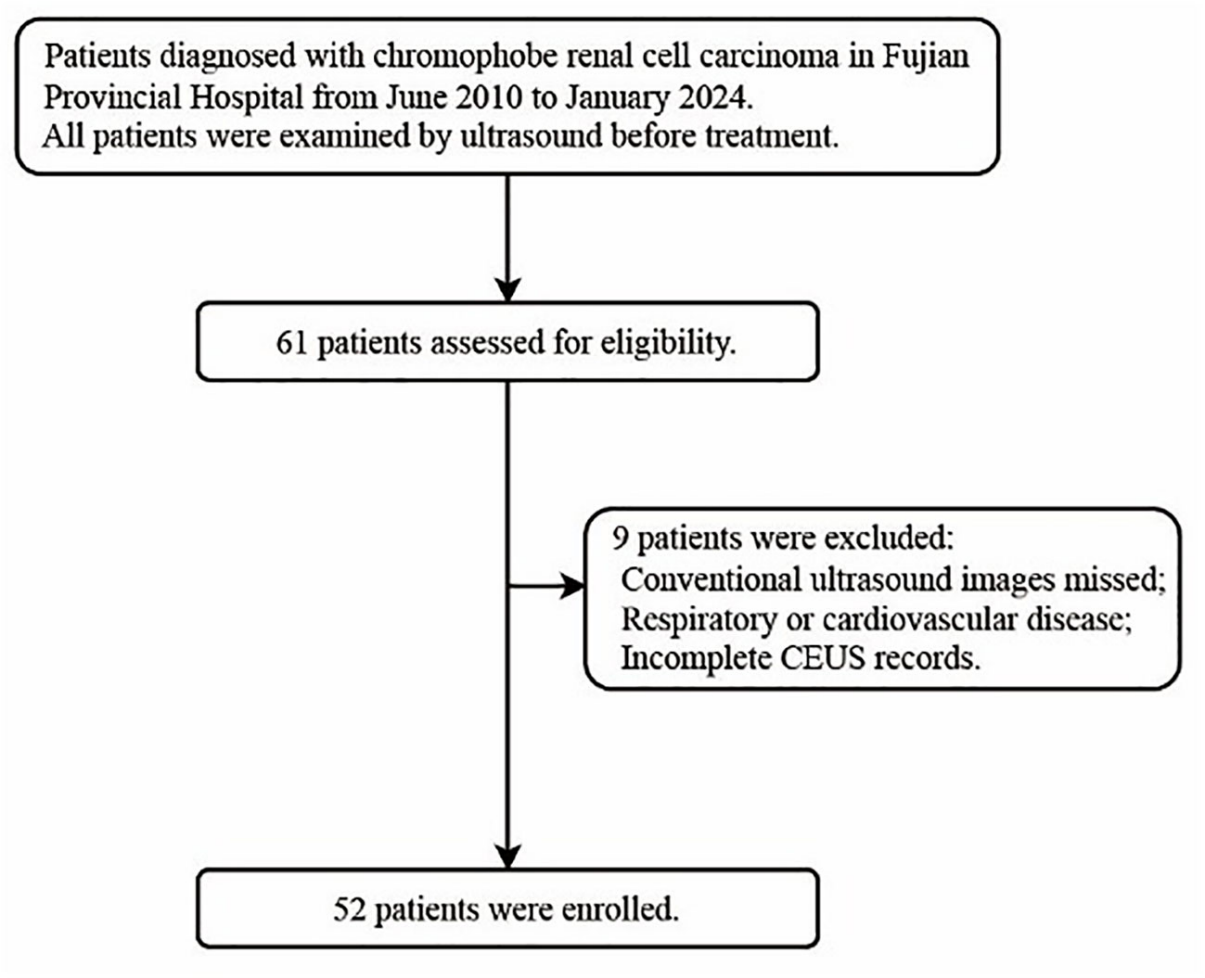
Flow diagram illustrating the inclusion and exclusion criteria adopted in this study.

The institutional review board approved this study (K-2024-10-020) and granted a waiver of informed consent for the review of records and ultrasound images. The inclusion and exclusion criteria are presented in the flowchart (**Figure 1**). Clinical data, including age, gender, initial symptoms, treatment approaches, and pathological staging, were extracted from electronic medical records.

### 2.2 Image acquisition

When multiple renal ultrasound images are available for review, priority should be given to those taken closest to the patient’s surgery. All scans were conducted using the EPIQ 7 ultrasound system (Philips, Netherlands), including conventional ultrasound, color Doppler imaging, and CEUS. Conventional ultrasound was used to assess tumor size, morphology, and echogenicity, while color Doppler imaging evaluated intratumoral vascularity. For each patient, continuous scanning was performed in both the coronal and transverse planes to evaluate the renal mass, and at least three representative coronal and three transverse images were subsequently acquired. CEUS was performed using SonoVue^®^ (Bracco, Italy). SonoVue was reconstituted with 5 ml of saline, and a 2.0 ml bolus was administered via the antecubital vein, followed by a 10 ml saline flush. Dynamic video acquisition started immediately after injection and was continuously recorded for 3 minutes. After the examination, cine loops were reviewed frame-by-frame, and representative images from the arterial phase, corticomedullary phase, late phase, and peak enhancement were selected for CEUS feature analysis. All images were selected by physicians with over 10 years of experience in renal ultrasound.

### 2.3 Image interpretation

Two ultrasound specialists, with 21 and 18 years of experience in CEUS imaging, independently evaluated all tumor imaging features, blinded to the clinical and pathological results. Any discrepancies in their assessments were resolved through consensus after consulting a third reader with 24 years of CEUS experience.

Coronal images demonstrating the maximal tumor cross-section along with adjacent renal cortex and renal sinus were preferentially selected for echo-intensity analysis. If appropriate coronal images were not available, axial images depicting the long axis of the tumor and surrounding normal renal parenchyma and sinus were used instead.

The selected images were then imported into 3D Slicer in DICOM format. Three regions of interest (ROIs)—the renal tumor, renal cortex, and renal sinus—were identified within the same image. The SliceRadiomics extension was utilized to extract the average grayscale intensity (echo intensity) for each region. To minimize the impact of grayscale intensity variation caused by different images and depths, the renal cortex and renal sinus were selected at the same depth, and the tumor-to-renal cortex echo intensity ratio was calculated for the image.

Conventional ultrasound imaging evaluates the following tumor parameters: location, shape, margins, orientation, tumor configuration, homogeneity, internal blood flow (grade 0: no blood flow; grade 1: 1–2 pixels of blood flow, usually < 1 mm in diameter; grade 2: 3–4 pixels or main blood vessels visible; grade 3: ≥5 pixels or ≥2 major blood vessels visible) (13), and the presence or absence of calcification and liquefactive necrosis.

The parameters and definitions for CEUS include: wash-in and wash-out (renal masses described as faster, slower, or synchronous with adjacent renal cortex perfusion), peak enhancement (degree of enhancement compared to surrounding renal tissue), homogeneous enhancement (complete enhancement without defects) or heterogeneous enhancement (presence of unenhanced areas), enhancement mode (peripheral to central, central to peripheral, or overall), perfusion defects (areas within tumors where no contrast agent entered due to ischemic necrosis) (14), and perilesional rim-like enhancement (defined as distinct rim-like enhancement around the tumor in the late phase) (15).

### 2.4 Statistical analysis

Statistical analyses were performed using SPSS software (version 26.0; IBM). Descriptive statistics for baseline variables were reported as means and standard deviations. For the tumor-to-renal cortex echo intensity ratio, which was not normally distributed, the data were summarized using the median and interquartile range (Q1, Q3). Boxplots were used to visually display the distribution, variability, and skewness of the ratio. Categorical variables were described as frequency counts and percentages.

## 3 Results

### 3.1 Patient demographics, clinical information, and pathology

The clinical data of the patients are summarized in **Table 1**. Among the patients, 75.0% (39/52) were incidentally diagnosed with tumors during routine physical examinations, while the remaining 25.0% (13/52) sought medical attention due to symptoms, primarily lower back pain and hematuria. Intraoperative pathological biopsies revealed that 75.0% (39/52) of patients were classified as T1 stage, and 17.3% (9/52) as T2 stage according to TNM staging. Treatment involved radical nephrectomy in 38.5% (20/52) of cases and partial nephrectomy in 61.5% (32/52).

**Table 1:** Patient demographics, clinical information, and pathologic findings for 52 patients with chRCC.

Characteristic	chRCC (n=52)
Sex	
Male	27 (51.9%)
Female	25 (48.1%)
Laterality	
Left	26 (50.0%)
Right	26 (50.0%)
Mean age, year	52.85 ± 13.34 (27-81)
Long diameter of RCCs, cm	5.06 ± 3.76 (1.29-15.60)
Symptom	
Incidental	39 (75.0%)
Symptomatic	13 (25.0%)
Operation	
Radical nephrectomy	20 (38.5%)
Partial nephrectomy	32 (61.5%)
Tumor Staging(T category)	
T1	39 (75.0%)
T2	9 (17.3%)
T3	4 (7.7%)
T4	0 (0%)

Values are expressed as the mean ± standard deviation or number (%).

### 3.2 Features of ultrasonic quantitative echo intensity values

**Figure 2:**
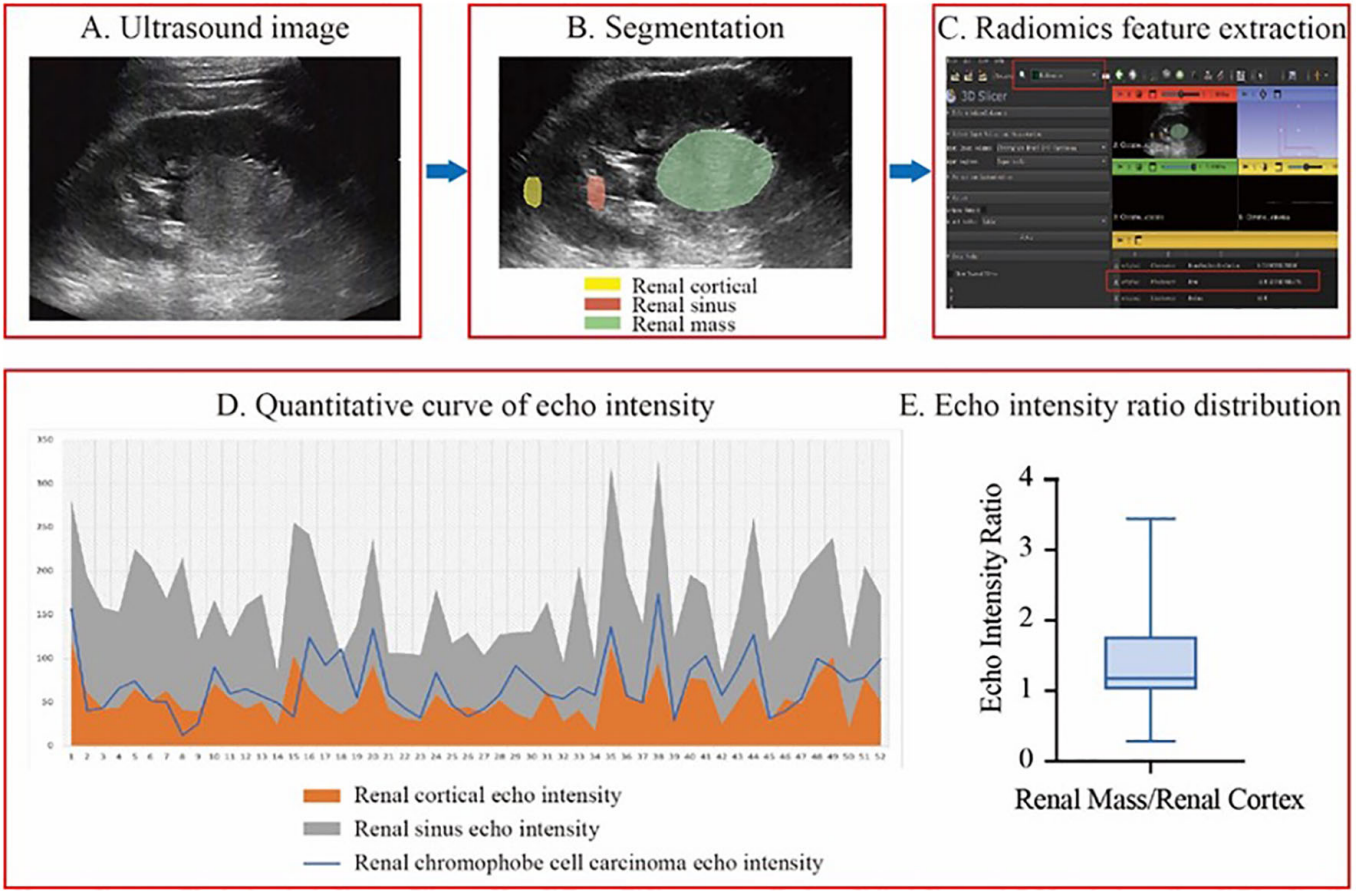
The workflow of echo intensity measurement and result analysis. **(A-C)**, the process of measuring echo intensity values. **(D)** A line graph of quantitative echo intensity values. **(E)** A box plot of the echo intensity ratio between renal masses and renal cortex from patients included in this study.

Only 23.1% (12/52) of renal chRCC tumors had echo intensity values lower than those of the renal cortex, and just 1.9% (1/52) showed values higher than those of the renal sinus. Meanwhile, 75.0% (39/52) had echo intensity values between the renal cortex and renal sinus, representing the majority of cases. The tumor-to-renal cortex echo intensity ratio ranged from 0.29 to 3.44, with an average of 1.41. And the median ratio was 1.18, with quartiles (Q1, Q3) of 1.01 and 1.78 (**Figure 2**).

### 3.3 Characteristics of conventional ultrasound in tumors

**Table 2** summarizes the conventional ultrasound characteristics of the tumors. Among the chRCC cases, 80.8% (42/52) of the tumors were exophytic, while 19.2% (10/52) were endophytic. Additionally, 80.8% (42/52) of the tumors had regular shape, and 90.4% (47/52) displayed clear tumor boundaries. In terms of composition, 78.9% (41/52) of the tumors were solid or predominantly solid, while 19.2% (10/52) exhibited a mixed cystic and solid structure. Regarding internal echoes, 26.9% (14/52) of the tumors showed homogeneous echoes, while 73.1% (38/52) had heterogeneous internal echoes. Moreover, calcification was observed in 19.2% (10/52) of the tumors, and liquefactive necrosis was present in 32.7% (17/52). Color Doppler ultrasound revealed that 32.7% (17/52) of chRCC cases had grade I internal blood flow signals, while 38.5% (20/52) exhibited grade II blood flow signals.

**Table 2:** Conventional ultrasound qualitative characteristics of chRCC.

Characteristic	chRCC (n=52)
Location	
Upper	12 (23.1%)
Middle	21 (40.4%)
Lower	19 (36.5%)
Shape	
Round/oval	42 (80.8%)
Irregular	10 (19.2%)
Margins	
Well defined	47 (90.4%)
Poorly defined	5 (9.6%)
Orientation	
Outward	42 (80.8%)
Inward	10 (19.2%)
Tumor configuration	
Cystic	1 (1.9%)
Solid	41 (78.9%)
Cystic with solid	10 (19.2%)
Homogeneity	
Homogeneous	14 (26.9%)
Heterogeneous	38 (73.1%)
Internal blood flow	
Grade: 0	2 (3.8%)
Grade: 1	17 (32.7%)
Grade: 2	20 (38.5%)
Grade: 3	13 (25.0%)
Calcification	
Absent	42 (80.8%)
Present	10 (19.2%)
Liquefactive necrosis	
Absent	35 (67.3%)
Present	17 (32.7%)

Values are expressed as the number (%).

### 3.4 Characteristics of CEUS in tumors

**Figure 3:**
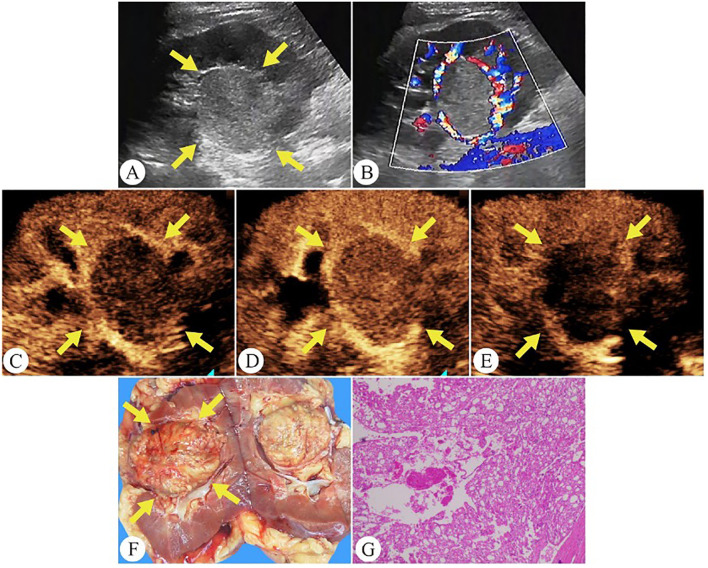
Chromophobe renal cell carcinoma in a 35-year-old man. **(A)** On grayscale ultrasound, a well-defined solid mass is observed in the right kidney (arrows). **(B)** Color Doppler flow imaging shows rich blood flow signals around the tumor periphery. **(C)** During the cortical phase of CEUS, the tumor exhibits a slow wash-in (arrows). **(D)** The tumor exhibits homogeneous peak enhancement and displays a perilesional rim-like enhancement on CEUS (arrows). **(E)** During the excretory phase of CEUS, the tumor exhibits a fast wash-out (arrows). **(F)** The gross specimen reveals the tumor as a pale yellow solid mass with a well-defined capsule (arrows). **(G)** Pathological HE-stained section (200× magnification).

**Table 3** outlines the CEUS features of chRCC. Among the tumors, 48.4% (15/31) demonstrated slow wash-in, 29% (9/31) had simultaneous wash-in, and 22.6% (7/31) showed fast wash-in. Additionally, 77.4% (24/31) of the tumors exhibited fast wash-out. 71.0% (22/31) of tumors displayed homogeneous enhancement. In terms of enhancement pattern, 64.5% (20/31) showed overall enhancement, while 32.3% (10/31) exhibited enhancement from the periphery to the center. At peak enhancement, 25.8% (8/31) of tumors showed hypoenhancement, 41.9% (13/31) showed isoenhancement, and 32.3% (10/31) demonstrated hyperenhancement. Finally, 19.4% (6/31) of tumors presented perfusion defects, and 61.3% (19/31) displayed PRE (**Figure 3**).

**Table 3:** CEUS qualitative characteristics of chRCC.

Characteristic	chRCC (n=31)
Wash-in	
Slow-in	15 (48.4%)
Simultaneous-in	9 (29.0%)
Fast-in	7 (22.6%)
Peak enhancement	
Hypoenhancement	8 (25.8%)
Isoenhancement	13 (41.9%)
Hyperenhancement	10 (32.3%)
Homogeneity	
Homogeneous	22 (71.0%)
Heterogeneous	9 (29.0%)
Enhancement pattern	
Peripheral to central	10 (32.3%)
Central to peripheral	1 (3.2%)
Overall	20 (64.5%)
Wash-out	
Slow-out	4 (12.9%)
Simultaneous-out	3 (9.7%)
Fast-out	24 (77.4%)
Perfusion defects	
Absent	25 (80.6%)
Present	6 (19.4%)
Perilesional rim-like enhancement	
Absent	12 (38.7%)
Present	19 (61.3%)

Values are expressed as the number (%).

## 4 Discussion

ChRCC originates from the distal and collecting tubules and is the third most common histological subtype in RCC (16, 17). Our study introduces two key innovations in analyzing its ultrasound characteristics. First, we pioneered the use of 3D Slicer software to quantitatively analyze the echo intensity values of renal tumor ultrasound images, enhancing objectivity and reproducibility in assessment. Second, our research comprises the largest cohort dedicated exclusively to defining the ultrasound features of chRCC, providing a more comprehensive characterization of its imaging presentation.

In this study, we measured the echo intensity values of the renal cortex and renal sinus at the same depth within the same ultrasound image and compared the echo intensity of the renal mass to these regions. This approach minimized the impact of grayscale differences across different images and depths within the same image. The results demonstrated that 75% of the mass echo values fell between those of the renal cortex and renal sinus, 23.1% were lower than the renal cortex, and only 1.9% were higher than the renal sinus. Additionally, we calculated the ratio of echo values between the renal masses and the renal cortex to standardize the results under different ultrasound gain settings. The echo value ratio ranged from 0.29 to 3.44, indicating considerable variability in tumor echo intensity. Median and quartile analyses revealed that the echo intensity of most renal chromophobe cell carcinomas was between 1% and 78% higher than that of the renal cortex, suggesting a consistent trend where the echo intensity of chRCC is typically higher than the renal cortex but lower than the renal sinus. These findings may provide valuable insights for non-invasive clinical diagnosis.

Intraoperative pathology revealed that 75.5% of patients had T1-stage tumors, while 17% were diagnosed at T2 stage. Compared to clear cell renal cell carcinoma (ccRCC) and papillary renal cell carcinoma (pRCC), chRCC exhibited a lower degree of invasion. John C. Cheville et al. found that 23% of pRCC cases are multifocal, while multifocality is observed in only 7% of ccRCC and 8% of chRCC cases (18). Similarly, Polascik et al. emphasized that bilateral and multifocal involvement is a distinctive feature of pRCC (19). In contrast, chRCC in our study predominantly presented as a solitary, solid mass with a regular shape, with no cases of multiple lesions observed. This finding is crucial for the differential diagnosis of chRCC.

**Figure 4:**
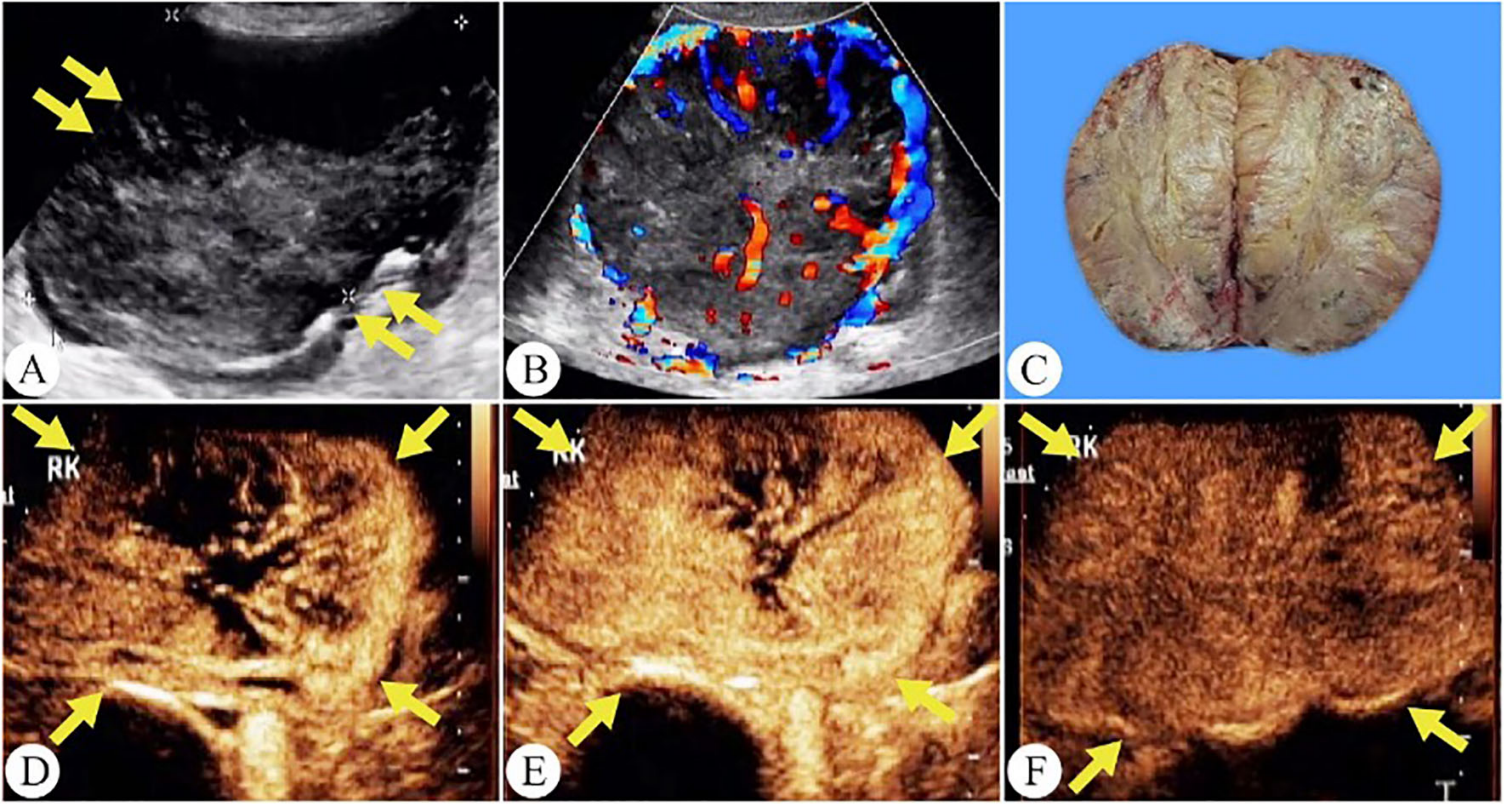
Chromophobe renal cell carcinoma in a 56-year-old woman. **(A)** On grayscale ultrasound, a solid mass is observed in the right kidney (arrows). **(B)** Color Doppler flow imaging reveals a spherical blood flow pattern. **(C)** The gross specimen shows a tumor with well-defined margins and a surrounding capsule. **(D)** The tumor shows a peripheral-to-central enhancement pattern on CEUS(arrows). **(E)** Peak hyperenhancement is observed in the tumor on CEUS. **(F)** During the excretory phase of CEUS, the tumor exhibits a fast wash-out (arrows).

On CEUS, 71% of chRCC tumors showed homogeneous enhancement. Heterogeneous enhancement, on the other hand, was associated with more aggressive tumor behavior and poorer clinical outcomes. Notably, the 5-year cancer-specific survival rate for chRCC was 60.7% in cases with necrosis and 94.0% in those without necrosis (20). Wu et al. reported that 20% of ccRCC, 46.6% of pRCC, and 54.9% of chRCC cases demonstrated PRE (9). In our study, PRE was observed in 61.3% of tumors, a higher proportion compared to previous findings (**Figure 4**). Among the common RCC subtypes, chRCC displayed a relatively higher frequency of PRE, providing key clues for differentiating between various renal tumor types.

However, our study has several limitations. First, the rarity of chRCC led to a relatively small sample size, potentially limiting the generalizability of our findings. Second, our quantitative analysis primarily concentrated on the echo intensity characteristics of chRCC. In future research, we plan to incorporate a comparative analysis of other renal tumor subtypes to enhance the broader applicability of our results.

In conclusion, this study demonstrates that combining conventional ultrasound features, CEUS characteristics, and quantitative echo-intensity analysis using 3D Slicer provides an objective and reproducible method for the imaging evaluation of chRCC. This multi-parametric, non-invasive approach may assist in the preoperative differentiation of RCC subtypes and support more informed clinical decision-making for individualized patient management.

## Data Availability

The original contributions presented in the study are included in the article/Supplementary Material. Further inquiries can be directed to the corresponding author.
